# Use of the Superficial Circumflex Iliac Artery Perforator Flap for Urethra and/or Shaft Reconstruction in Gender-Diverse Persons: 10-Year Single-Center Experience

**DOI:** 10.1097/PRS.0000000000011830

**Published:** 2024-10-28

**Authors:** Anouk De Gelder, Danny Young-Afat, Karel Claes, Shane Morrison, Nikolaas Lumen, Anne-Françoise Spinoit, Mieke Waterschoot, Salvatore D’Arpa, Stan Monstrey, Marlon Buncamper

**Affiliations:** Ghent, Belgium; Amsterdam, the Netherlands; Seattle, WA; and Palermo, Italy; From the Departments of 1 Plastic Surgery; 4 Urology, Ghent University Hospital; 2 Department of Plastic Surgery; 3 Division of Plastic Surgery, Department of Surgery, University of Washington Medical Center and Seattle Children’s Hospital; 5 Plastic and Reconstructive Surgery, Villa Serena Clinic.

## Abstract

**Background:**

Although the free radial forearm flap (FRFF) is the standard for phalloplasty, the conspicuous donor-site scar, need for microsurgery, and tendency for the phallus to deflate over time leads both patients and surgeons to seek alternatives. The authors describe their long-term experience with the pedicled superficial circumflex iliac artery (SCIP) flap for urethral or penile shaft reconstruction, or both. This flap can be applied for similar indications as FRFF phalloplasty, but with primary closure of the donor sites and without microsurgery.

**Methods:**

The authors retrospectively reviewed surgical outcomes of all patients who underwent urethra or shaft reconstruction, or both, using SCIP flaps as part of phalloplasty for gender-affirming surgery in their center between 2012 and 2022.

**Results:**

Over a 10-year period, 55 SCIP flaps were performed as part of phalloplasty. This included 47 unilateral SCIP flaps, 10 of which were used for shaft reconstructions and 37 for urethra reconstructions. Primary closure was achieved in 100% of patients. No failures were observed for shaft reconstructions. For urethra reconstructions, 8 SCIP urethras (14%) failed completely, and 3 SCIP flaps were converted to free flaps. In total, 82% of patients with a SCIP urethra reconstruction were able to stand while voiding. Eight bilateral SCIP flaps were performed for 1-stage shaft and urethra creation; among these, 3 patients (38%) experienced urethral fistulas or strictures.

**Conclusions:**

This study shows that the SCIP flap is a technically feasible and safe pedicled alternative for urethral and penile shaft reconstruction in gender-diverse individuals, with similar urethral complication rates compared with FRFF and anterolateral thigh flap phalloplasty, but with potentially lower shaft sensation.

**CLINICAL QUESTION/LEVEL OF EVIDENCE:**

Therapeutic, IV.

Phallic reconstruction can be completed with a wide array of pedicled and free flaps from various donor sites. The ideal phallic reconstruction should allow for voiding while standing, sensation (tactile and erogenous), sexual satisfaction, low morbidity for both recipient and donor sites, and an aesthetically satisfying phallus.^1^ The literature suggests that the free radial forearm flap (FRFF) is the most frequently applied flap, followed by the pedicled anterolateral thigh (ALT) flap.^2^ The FRFF is considered the standard because of its reliable vascularity; successful sensible innervation using the medial, lateral, and more recently posterior antebrachial cutaneous nerve as recipient nerves; and thin tissue, allowing for reconstruction of both phallus and urethra in 1 stage.^3–13^ However, its requirement for microsurgical anastomosis comes with inherent free-flap–related risks and adds to the operative time and difficulty. In addition, its conspicuous donor-site scar and its tendency to deflate over time are important consequences to take into account. As a result, less complex pedicled alternatives with similar or lower complication rates are needed.

Our previous experience^14^ showed an increasing interest in the pedicled ALT flap for phalloplasty. However, because of the thickness of ALT, multiple secondary corrections are generally required to achieve an aesthetically pleasing result. Furthermore, a tube-in-tube technique can rarely be achieved for urethra reconstruction due to subcutaneous thickness (>1 cm).^3,7,15^ Hence, a second flap is then needed for urethral reconstruction.

A promising alternative is the pedicled superficial circumflex iliac artery perforator (SCIP) flap, which was first described by Koshima et al.^16^ for penile reconstruction. Potential advantages include the ability to perform the phalloplasty without microsurgery and a donor site that is closed primarily and hidden under clothes. However, a known disadvantage is the slow recovery of sensibility compared with the FRFF.^17–19^ Moreover, to allow for primary closure of the donor site, the SCIP flap does not allow for a tube-in-tube design. Instead, bilateral pedicled SCIP flaps can be used, where one side is used for urethral reconstruction and the other side is used to create the phallic shaft.

The SCIP flap is increasingly used for phalloplasty in our center, based on both patient and surgeon preferences. We present our 10-year experience and long-term outcomes of pedicled SCIP flaps for all genital gender-affirming indications for which it has been applied (ie, urethral or phallic shaft reconstruction, or both).

## PATIENTS AND METHODS

### Eligibility Criteria

All patients diagnosed with gender dysphoria who underwent a urethra or shaft reconstruction, or both, as part of phalloplasty using unilateral or bilateral SCIP flaps between 2012 and 2022 were included in this study. We obtained approval from the ethics committee of UZ Ghent (ONZ-2022-0537). All individuals included met World Professional Association of Transgender Health Standards of Care criteria at the time of considering surgery.^20^ We only performed surgery on nonsmokers or after smoking cessation of at least 3 months. In addition, based on standard local protocol, all patients had to have undergone colpectomy or hysterectomy at least 1 year in advance of surgery.

Indications for using SCIP flaps were one or more of the following: preference for an inguinal or flank scar over other donor-site options; pinch test in the inguinal or flank area of less than 1.0 cm; technical unsuitability for FRFF or ALT (eg, vascular insufficiency based on Allen test, pinch test of the lateral thigh of more than 1.5 cm, absence of an adequate perforator on angiographic computed tomography of the anterolateral thigh area); failed primary phalloplasty or the need for a second flap; and acceptance of potentially lower sensation compared with FRFF and ALT options.

### Surgical Procedure

All procedures were performed separately by the same 2 surgeons (M.B. and S.D.). At the time of consultation, the surgeons explained all available techniques and the advantages and disadvantages of each technique for which the patient seemed eligible, including potentially lower sensation of the SCIP flap compared with FRFF and ALT flaps. Gender-diverse individuals who met the abovementioned eligibility criteria were considered candidates for a SCIP flap phalloplasty.

**Fig. 1.**
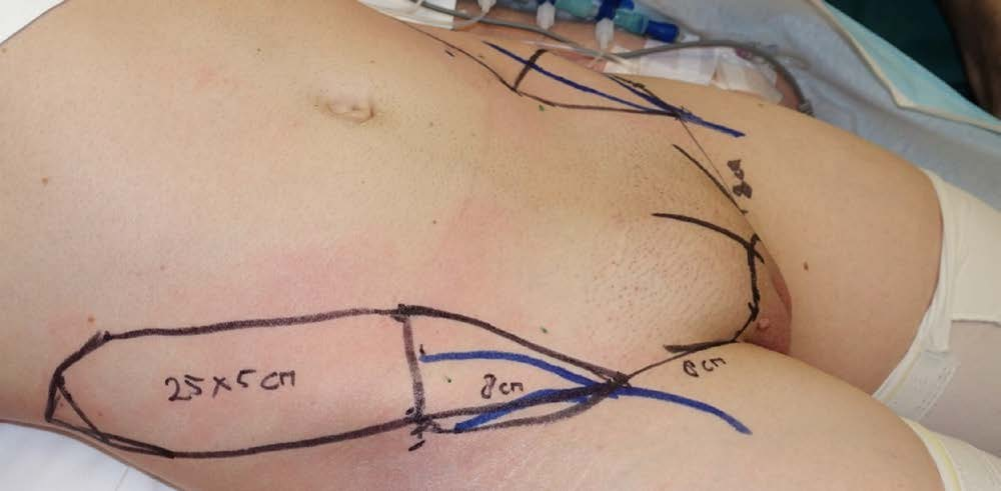
Overview of preoperative design for a double-sided SCIP flap for urethra and penile shaft reconstruction. The *blue lines* indicate the superficial branch (on the medial side) and deep branch (on the lateral side) of the superficial circumflex iliac artery followed by hand-held Doppler.

In primary cases, the recipient site was prepared by the urology team. When indicated, the urologist performed the vaginectomy, urethral lengthening, and harvesting of the dorsal clitoral nerve. In general, when used for urethral reconstruction, we aimed for the SCIP flap to have a width of at least 4 cm to allow for tension-free tubularization around a urinary catheter (Fig. 1). If a bilateral SCIP flap was performed, 1 flap was used to reconstruct the urethra, and the second flap was wrapped around the reconstructed urethra as a shaft. The maximum width of a second SCIP flap was designed according to a pinch test, aiming to allow for primary closure of the donor site. All donor sites were closed primarily. We aim for a minimum of 12 to 14 cm in penile length, not including the distal elliptical extension to allow for easy closure of the donor site and the proximal part that will be buried under the inguinal skin after rotation of the flap. The total length of the skin island is usually between 23 and 24 cm (Fig. 1). (**See Figure, Supplemental Digital Content 1**, which shows design for a SCIP flap for urethra reconstruction. The total length of the flap is measured at 25 cm. From the origin of the perforator, being the point of rotation, up to the start of the actual urethral flap, we measured 8 cm. This proximal part of the flap will be buried under inguinal skin after actual rotation of the flap has taken place. The most distal elliptical part of the flap [around 2 cm] allows for easy closure of the donor site, but will be excised after harvesting of the flap. The total urethral length will eventually be around 15 cm. A width of at least 4 cm is mandatory for tubularization around a urinary catheter. In this case, the width was 5 cm, http://links.lww.com/PRS/H684. **See Figure, Supplemental Digital Content 2**, which shows design for a SCIP flap for penile shaft reconstruction. The total length of the flap is measured at 27 cm. Same as with the urethral flap, from the origin of the perforator, being the point of rotation, up to the beginning of the actual flap, we measured 8 cm. This proximal part of the flap will be buried under inguinal skin after actual rotation has taken place. The most distal elliptical part of the flap allows for easy closure of the donor site, but will be excised after harvest. The total penile length will eventually be around 15 cm in this case. The width of the flap is 9 cm, which still allows us to easily close the donor-site primarily, http://links.lww.com/PRS/H685. **See Figure, Supplemental Digital Content 3**, which shows an overview of the preoperative design for a unilateral SCIP flap for penile shaft reconstruction. The medial *dotted square* indicates subcutaneous undermining to provide additional bulk, http://links.lww.com/PRS/H686.)

We aim to include the anterior division of the lateral cutaneous nerve branch of thoracic 9th, 10th, or 11th intercostal nerves in the design for coaptation to the dorsal clitoral nerve for the shaft reconstruction.^21^ These branches can be found longitudinally along the midaxillary line. We follow the nerves as far as possible, creating enough length for tension-free nerve coaptation, without creating additional donor-site morbidity. In all cases, irrespective of the flap used, we aim for 1-stage procedures to provide final results immediately. In our center, phalloplasty is never delayed unless there is an unexpected problematic vascular supply during the initial procedure, which has only occurred in 1 flap over a 10-year period.

According to local protocol, the patient remains on bed rest for 5 days, after which they may start mobilizing, and returns home on the postoperative day 7, unless complications occur.

### Follow-Up

**Fig. 2.**
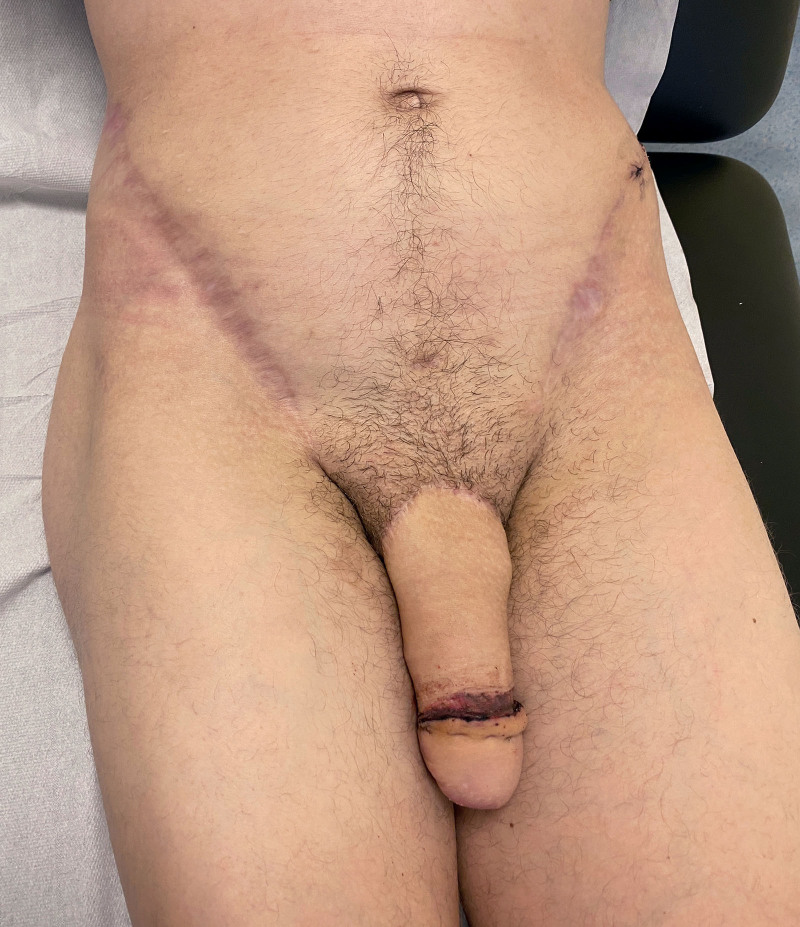
Long-term (6 months) postoperative result for a bilateral SCIP flap. The coronaplasty was performed 2 weeks before this photograph was taken and is in the healing phase. We used the left-sided scar as a full-thickness skin graft to perform the coronaplasty.

The urethral catheter was routinely removed after 3 weeks, and the suprapubic cystostomy catheter was taken out when residual urine was below 100 mL. Uroflowmetry or urethrography is performed routinely. In shaft-only cases, a urethrostomy is made, and no suprapubic catheter is placed. The urethrostomy catheter is removed after 7 days. If desired, coronaplasty can be performed several months after the operation (Fig. 2). Our technique for coronaplasty has been described in greater detail in a previous article,^22^ including short-term preliminary results of this technique. Longer-term postoperative results are shown in Figures 2 through 6.

**Fig. 3.**
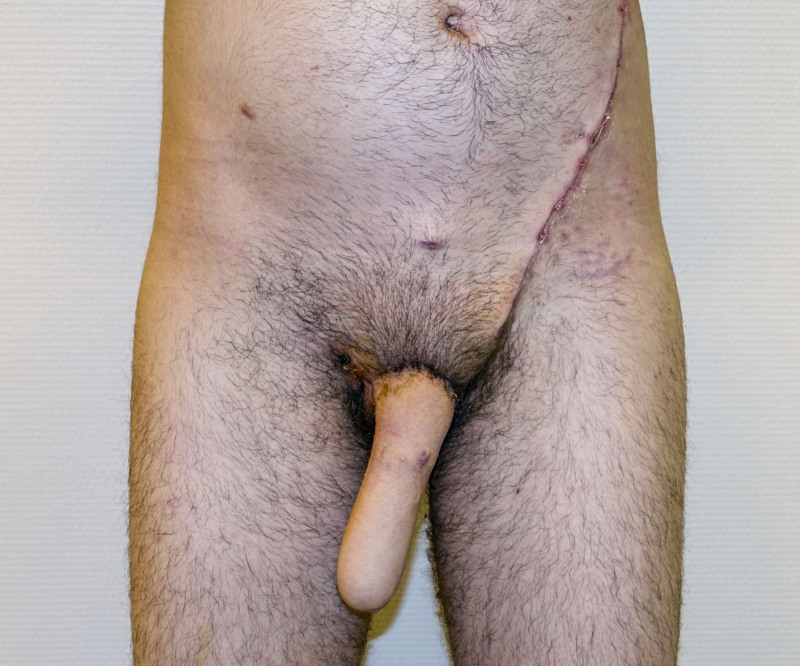
Frontal view of a unilateral SCIP flap for penile shaft reconstruction 3 months postoperatively.

**Fig. 4.**
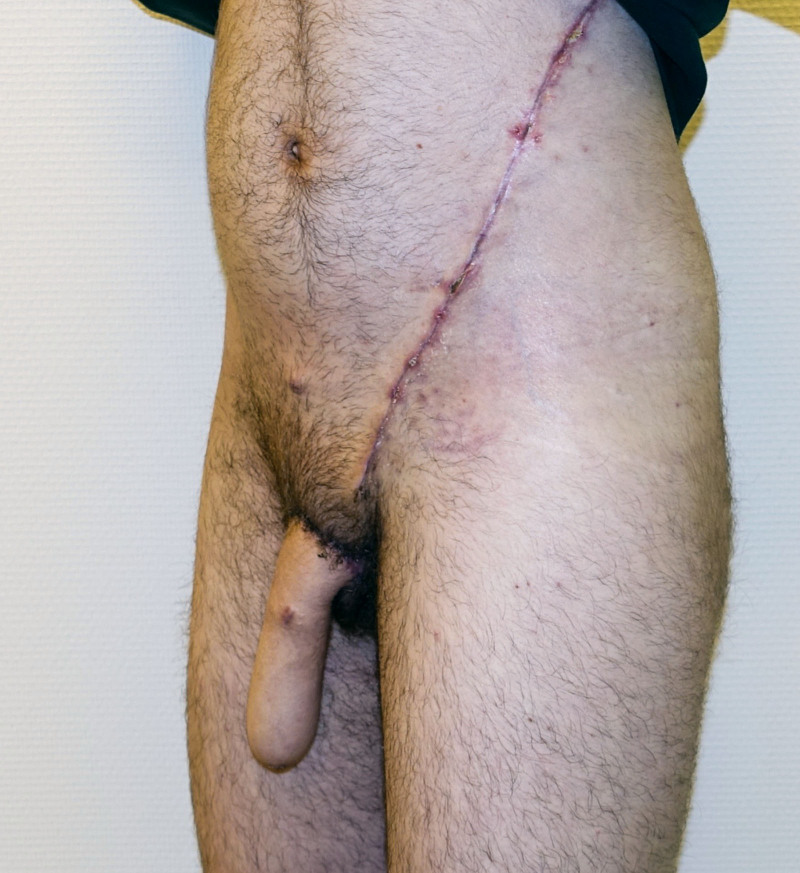
Side view of a unilateral SCIP flap for penile shaft reconstruction 3 months postoperatively.

**Fig. 5.**
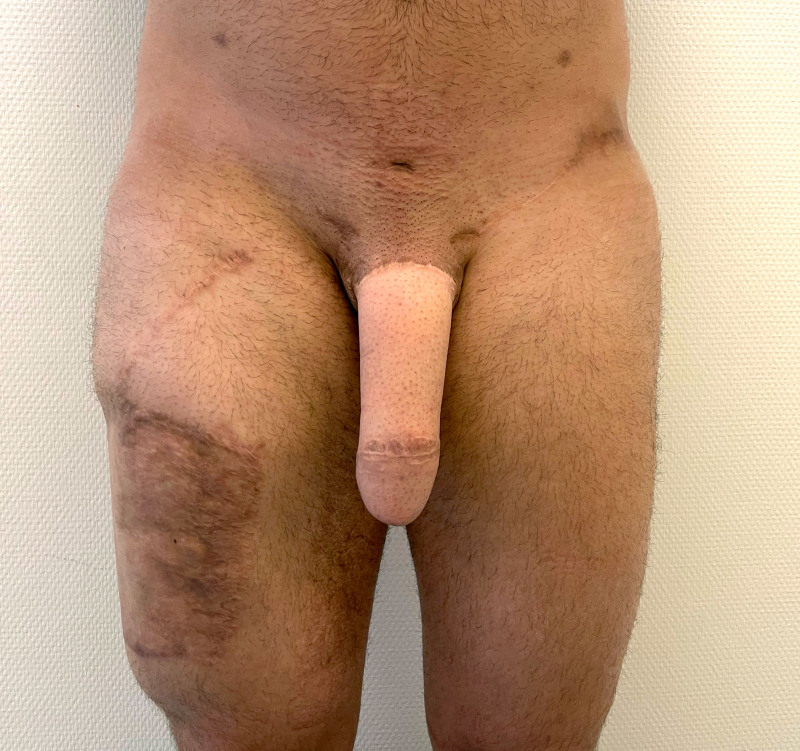
Long-term (18 months) postoperative result for ALT shaft with SCIP urethra, after having received erectile prothesis. Frontal view.

**Fig. 6.**
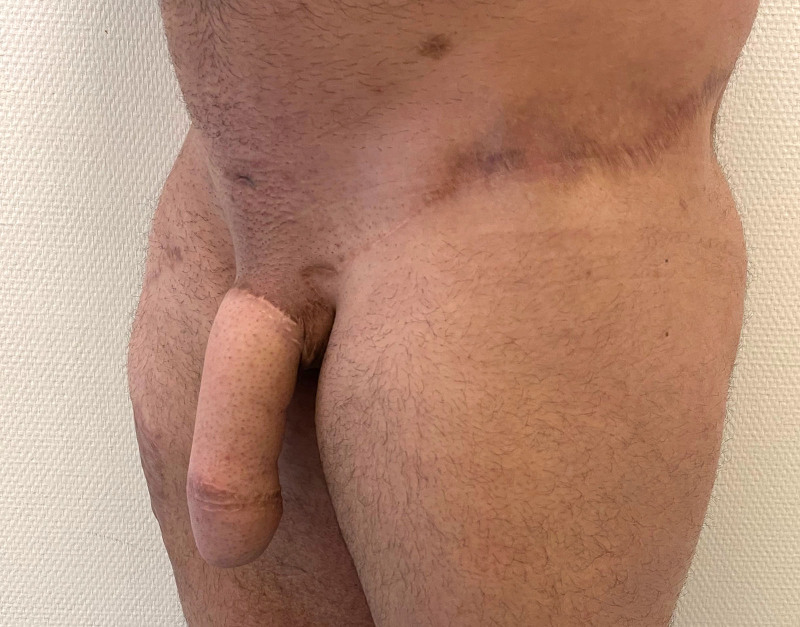
Long-term (18 months) postoperative result for ALT shaft with SCIP urethra, after having received erectile prothesis. Side view.

### Data Collection

All patients meeting study eligibility criteria were analyzed. Their data were anonymized and collected in a secured electronic database by 2 members of the research team (A.D.G. and K.C.), and included patient demographics, phallic reconstruction type and indications, need for additional surgical procedures, date of the last clinical evaluation, and major postoperative complications (ie, requiring surgical reintervention).

## RESULTS

During a 10-year period, 55 gender-diverse individuals underwent a urethral or penile shaft reconstruction, or both, with a SCIP flap (Table 1). Their median age at the time of surgery was 24 years (range, 19 to 53 years), and the median body mass index was 21.2 (range, 16.7 to 31.8). Patients underwent at least 12 continuous months of hormone therapy as appropriate to the patient’s gender goals. In this study, all patients were under hormonal treatment. We saw no significant comorbidities in any of the included patients (eg, diabetes, cardiovascular comorbidities). Forty-seven patients received a unilateral SCIP flap and 8 received a bilateral SCIP flap. With exception of 4 patients, who received the SCIP flap as a secondary flap after initial reconstructive failure, a concomitant vaginectomy was performed in all patients. The median surgery time was 460 minutes (range, 135 to 764 minutes). Thirty-six patients needed, on average, 2 (range, 1 to 6) additional surgical interventions for débridement, shape correction, or a new flap after failure. Twenty-five patients needed at least 1 extra urologic intervention after the initial procedure (average, 2; range, 1 to 6). The average hospital stay was 8 days (range, 7 to 15 days). The mean follow-up time was 26 months (range, 1 to 97 months). Three SCIP flaps had to be converted to free flaps because of insufficient pedicle length, which in all cases involved a SCIP flap used for urethral reconstruction.

**Table 1.** Participant Characteristics^a^

Characteristics	Values
Age at time of surgery, yrs, mean (range)	24 (19–53)
Body mass index, mean (range)	21.2 (16.7–31.8)
Hysterectomy, no. (%)	55 (100)
Hormonal treatment, no. (%)	55 (100)
Surgery time, min, mean (range)	460 (135–764)
Admission days in hospital, average (range)	8 (7–15)
No. of additional surgical interventions (plastic surgery) in 36 patients, average (range)	2 (1–6)
No. of additional surgical interventions (urology) in 25 patients, average (range)	2 (1–6)
Follow-up time, mo, mean (range)	26 (1–97)

a All participants were transgender men. None had diabetes, cardiovascular disease, or a clotting disorder.

### SCIP Flap for Penile Shaft Reconstruction

Ten patients received a unilateral SCIP flap for shaft-only reconstruction. The procedure was performed as described above. All SCIP flaps for penile shaft creation were pedicled.

Five patients received a shaft-only phalloplasty without urethral lengthening with an immediate perineal urethrostomy.

The other 5 patients received a unilateral SCIP flap for shaft reconstruction, combined with FRFF for urethral lengthening, because the expected girth of 2 SCIP flaps would exceed functional penetration in these specific cases. The FRFF was performed with microsurgical end-to-side anastomosis of the radial artery to the femoral artery and end-to-end anastomosis of the cephalic vein to the saphenous vein. Two patients (20%) experienced a hematoma in the groin area, without clinical consequences, and 1 SCIP flap was partially lost at the tip due to insufficient vascularization (10%). No other complications were observed.

In 7 out of 10 patients with a shaft reconstruction, information on sensation was obtained. In 4 out of 7 patients (60%), sensation over the proximal part of the phallus was present, and 3 patients (40%) reported no sensation at all at time of the last follow-up, which in all cases entailed a short postoperative follow-up. Range of follow-up for these patients was between 3 and 25 months, so some additional sensation can be expected over time.

### SCIP Flap for Urethra Reconstruction

In 37 patients, the SCIP flap was used for urethra reconstruction, in combination with an ALT flap for the shaft. The ALT shaft was performed as described in an earlier article from our department.^14^ Tension-free tubularization of the ALT flap is paramount to avoid constriction of the SCIP urethra, and thus, was only offered to people who met criteria described above. Body mass index was not an exclusion criterion, because muscular body habitus or fat distributions that are not centered around the waist may still provide a pinch of 1 cm at the SCIP donor site.

In 4 cases, the SCIP flap was used for secondary reconstruction after failure of the initial FRFF urethra. One of these patients had a body mass index of 32 and a pinch above 1 cm, but because the SCIP flap was used as additional cover after (partial) FRFF urethra failure and dehiscence, this was of no objective.

Fourteen patients (38%) experienced urethral fistulas or strictures needing surgical intervention. There were 6 complete losses of the SCIP urethra (16%) and 4 partial losses (11%). Of these complete failures, 3 involved cases that were converted to a free flap due to insufficient pedicle length and 1 flap was delayed. All complete failures received a secondary flap, usually with an FRFF. Out of all patients receiving a SCIP urethra, 32 patients (89%) were able to void while standing once all interventions were completed.

### SCIP Flaps for Shaft and Urethra Reconstruction

Eight patients received a bilateral SCIP flap to create both shaft and urethra reconstruction in 1 stage. Of those, 3 patients (38%) experienced urethral fistulas or strictures. Two SCIP urethras (25%) were lost completely; 1 of these was surgically removed due to the patient’s preference, and a salvage perineal urethrostomy was performed in both of these patients without attempting a new flap, based on the patients’ preferences. Two other complications requiring surgical revision were noted, 1 due to bleeding in the vaginectomy surgical site and the other due to phallic wound dehiscence. Two patients reported sensation at the proximal part of the phallus; 1 patient had no sensation; and in 5 patients, the sensation data were not available. Range of follow-up for these patients was between 3 and 23 months, so some additional sensation can be expected (Table 2).

**Table 2.** SCIP Characteristics

Characteristics	SCIP Shaft		SCIP Urethra		Bilateral SCIP
	SCIP (S)	SCIP (S) + FRFF (U)	Secondary SCIP (U)	ALT (S) + SCIP (U)	
No. of patients	5	5	4	33	8
Operative time, min, mean (range)	287 (233–490)	431.2 (358–490)	190 (135–320)	492.8 (189–746)	377 (265–605)
Urethral complications of SCIP, *n* (%)	NA	NA	2 (50)	12 (36)	3 (37.5)
Other complications, *n* (%)	1 (20)	1 (20)	1 (25)	4 (12)	2 (25)
Conversion free flap/delay, *n* (%)	0/0	0/0	1 (25)/0	2 (0.6)/2 (0.6)	0/0
Partial/complete SCIP loss, *n* (%)	0/0	1 (20)/0	0/1 (25)	4 (12)/5 (15)	0/2 (25)
Average total no. of procedures (including extra urologic interventions)	1.8	2.8	4.5	3.6	2.5
Average additional plastic procedures	0.2	0.4	2	1.7	0.2
Sensation, *n* (%)	2 (40)	2 (20)	NA	NA	2 (25)
Void standing up, *n* (%)	NA	2 (20)	3 (75)	29 (87.9)	5 (62.5)

S, shaft reconstruction; U, urethral reconstruction.

## DISCUSSION

The SCIP flap is increasingly used for phalloplasty in our center, based on both patient and surgeon preference. We present our 10-year experience and long-term outcomes of pedicled SCIP flaps for phalloplasty for all applied indications (ie, urethral reconstruction, shaft reconstruction, or both).

In the ideal setting, phalloplasty provides an aesthetically pleasing phallus, retains erogenous and tactile sensation, enables voiding while standing, and allows for penetrative sexual intercourse.^1,23^ Numerous techniques have been developed, but the creation of a fully functional phallus remains elusive.^2,15,24,25^ To date, there are still no suitable replacements for erectile and urethral tissue, apart from erectile devices and autologous flaps.^15^ Each technique involves specific risks or drawbacks, including the large size of the flap needed for phallic tubularization. Such large flaps make them susceptible to vascular insults, which can be especially evident in free flaps, necessitating more reliable pedicled options.

A detailed breakdown of all flaps performed in our center is beyond the scope of this study. However, between 2004 and 2022, a total of 350 phalloplasty procedures were performed. Of these, 50% were FRFF phalloplasty with or without urethra lengthening. Roughly 40% were ALT shafts with or without urethra lengthening using another flap. The FRFF is the most performed flap in our center, but there has been an increase in the use of the SCIP flap for gender-affirming surgery, based both on patient and surgeon preference.

In this series, we reviewed 55 pedicled SCIP phalloplasty procedures over a 10-year period in one of the largest centers in Europe for gender-affirming surgery. We observed 8 complete losses of SCIP flaps (14%), which all involved using the SCIP flap for urethral reconstruction. This suggests that this flap may be too bulky and thus prone to vascular compromise after tubularization and compression from a composite thick flap (such as ALT) when wrapped around the SCIP. A similar experience has also been described by Heston and colleagues.^2^ As a result, as of December of 2019, we have moved away from using the SCIP–ALT combination. Nowadays, when an ALT shaft with urethra lengthening is chosen by a patient, we usually suggest FRFF urethra unless the patient specifically requests a SCIP urethra and has the body habitus to receive a bilateral SCIP flap. Adequate patient selection is of utmost importance to decrease the chances for venous congestion. Bulk is minimized by strictly adhering to the 1-cm pinch test at the level of the donor site for the SCIP flaps. If needed to allow for tension-free tubulization, and thus reducing chances for venous congestion, we temporarily use split thickness on the ventral side of the phallus.

In general, urethral lengthening is the most complication-generating part of phalloplasty. We found complications after urethra reconstruction, in the form of fistulas or strictures, in 38% of our patients. Most urethral fistulas (57%) were at the anastomotic site, which is also the junction of the distal and most vascular compromised aspect of urethral and labial flaps involved in this procedure.

A large array of urethral reconstruction techniques have been reported, indicating that there is no consensus on which one is the best when a tube-in-tube reconstruction cannot be used. In our center, we opt for a second flap when a tube-in-tube reconstruction cannot be used, and we do not use skin grafts because of the very low success rates in this setting.

Despite the high rates of urethral strictures and fistulas for all these techniques, many patients still request urethral lengthening, hoping to void while standing.^14^ A tube-in-tube design is not possible for SCIP flaps when aiming for primary closure of the donor site, but the bilateral SCIP flap provides an elegant alternative. Furthermore, the design allows for selective exclusion of innervation on the urethral flap, thus avoiding troublesome sensation in the neourethra. We nowadays apply unilateral SCIP flaps for shaft-only reconstructions, and double-sided SCIP flaps for combined shaft and urethra reconstructions.

Our reported urethral complication rates are within range of the rates reported in our previous articles on urethral reconstruction in ALT flap phalloplasty (33% to 53%) and tube-in-tube FRFF (41%).^14,26–28^ When we compare the groups, we see comparable urethral complication rates in the SCIP shaft with FRFF urethra (40%) versus the bilateral SCIP (38%) and the ALT shaft with SCIP urethra (36%). However, given the uneven group distribution, it is difficult to draw generalizable conclusions.

Literature review suggests the SCIP flap is rarely used despite its minimal donor-site morbidity and avoidance of microsurgery. Some reported disadvantages that preclude use of the SCIP were low sensation, atrophy over time, limited ability to urinate in the standing position, and inability to engage in penetrative sexual intercourse.^17–19^

By making specific alterations to the SCIP flap for phalloplasty, we have aimed to address some of the concerns. Inclusion of thoracic intercostal 9th, 10th, or 11th nerves for coaptation theoretically allows for tactile sensation, and burial of the denuated clitoris can allow for erogenous sensation. A literature review by Morrison et al.^29^ showed some recovered glans sensitivity in 90% and erogenous sensation in more than 95% of cases, using the lateral and medial antebrachial cutaneous and lateral femoral cutaneous as the most common recipient nerves.

Data on sensation in SCIP phalloplasty are limited in the literature, as well as in our study. The SCIP flaps that were performed before December of 2019 were all urethral or salvage flaps, where no sensation was needed, and thus no evaluation of sensory outcomes was performed. In clinic, we always discuss the matter of sensation with the patient before surgery. During the preoperative shared decision-making process, it is clearly mentioned that the SCIP flap provides the least tactile sensation out of the 3 options we offer (FRFF more than ALT more than SCIP). Therefore, we primarily advise the FRFF for individuals who unequivocally desire an erectile device, for whom a fully sensate phallus is required before implantation of the device. Therefore, the FRFF remains the standard in our center for patients with a strong desire for erectile devices.

Because SCIP shafts became available in our center more recently, they have less than 2 years follow-up, and therefore, the point where erectile devices can be inserted has not been reached. To date, 2 of the patients receiving shaft-only procedures report sensation in up to three fourths of the phallus after 18 months. Sensation may reach the top of the phallus over time, but we expect sensation to remain lower than what FRFF and ALT flaps can provide. Once adequate follow-up has been reached, we will report results for SCIP shaft phalloplasty.

To assess sensation, we plan to use GENDER-Q in future studies.^30^ The GENDER-Q will allow for robust evaluation of clinically relevant patient-reported outcomes, once this instrument has been validated and released.

This study has several strengths and limitations. To date, this is one of the largest series with long-term follow-up after SCIP phalloplasty. Nonetheless, the sample size of this study is relatively small, and important limitations inherent to the retrospective nature of this study (eg, selection bias) were present. Despite positive surgical results, there is a strong need for better evaluation of the patients’ perspectives, as well as more uniform outcome selection in this field. Future research is needed comparing long-term results in large representative cohorts of patients undergoing SCIP phalloplasty with patients undergoing other available techniques. Nonetheless, for phalloplasty, a one-size-fits-all option will never be found, and patient selection based on anatomy and personal preference will remain the most important factor when deciding on the final treatment plan.

We would like to emphasize that the SCIP flap is not a superior choice in our center. We merely wish to add the SCIP flap as an option for phallic reconstruction, as different patients have different wishes for their preferred donor site and outcomes. Therefore, given our current results, we believe that the SCIP flap may be added to the available options when considering phalloplasty.

## CONCLUSIONS

Pedicled SCIP flap phalloplasty is gaining popularity in patients who want to avoid the scars associated with FRFF or ALT phalloplasty. This study shows that the SCIP flap is a technically feasible and safe alternative for urethral and phallic shaft reconstruction in gender-diverse individuals, with similar urethral complication rates compared with FRFF and ALT phalloplasty; however, sensation appears to be lower.

## DISCLOSURE

The authors have no financial relationships or conflicts of interest to report.

## Supplementary Material







## References

[1] Monstrey S Hoebeke P Selvaggi G. Penile reconstruction: is the radial forearm flap really the standard technique? Plast Reconstr Surg. 2009;124:510–518. 19644267 10.1097/PRS.0b013e3181aeeb06

[2] Heston AL Esmonde NO Dugi DD 3rd Berli JU. Phalloplasty: techniques and outcomes. Transl Androl Urol. 2019;8:254–265. 31380232 10.21037/tau.2019.05.05 PMC6626313

[3] Mutaf M Isik D Bulut O Büyükgüral B. A true nonmicrosurgical technique for total phallic reconstruction. Ann Plast Surg. 2006;57:100–106. 16799318 10.1097/01.sap.0000208991.22264.b5

[4] Lumen N Monstrey S Selvaggi G. Phalloplasty: a valuable treatment for males with penile insufficiency. Urology. 2008;71:272–276. 18308099 10.1016/j.urology.2007.08.066

[5] Lumen N Monstrey S Ceulemans P van Laecke E Hoebeke P. Reconstructive surgery for severe penile inadequacy: phalloplasty with a free radial forearm flap or a pedicled anterolateral thigh flap. Adv Urol. 2008;2008:1–5. 10.1155/2008/704343 PMC2581740 19009034

[6] Descamps MJ Hayes PM Hudson DA. Phalloplasty in complete aphallia: pedicled anterolateral thigh flap. J Plast Reconstr Aesthet Surg. 2009;62:e51–e54. 17574944 10.1016/j.bjps.2007.04.014

[7] Lee GK Lim AF Bird ET. A novel single-flap technique for total penile reconstruction: the pedicled anterolateral thigh flap. Plast Reconstr Surg. 2009;124:163–166. 19568056 10.1097/PRS.0b013e3181ab2593

[8] Rubino C Figus A Dessy LA. Innervated island pedicled anterolateral thigh flap for neo-phallic reconstruction in female-to-male transsexuals. J Plast Reconstr Aesthet Surg. 2009;62:e45–e49. 18455975 10.1016/j.bjps.2007.11.056

[9] Rashid M Aslam A Malik S Tamimy MS Ehtesham-ul-Haq Aman S Jamy O. Clinical applications of the pedicled anterolateral thigh flap in penile reconstruction. J Plast Reconstr Aesthet Surg. 2011;64:1075–1081. 21324765 10.1016/j.bjps.2011.01.009

[10] Holzbach T Giunta RE Machens HG Müller D. Phalloplasty with pedicled anterolateral thigh flap (“ALT-flap”) [in German]. Handchir Mikrochir Plast Chir. 2011;43:227–31. 21495001 10.1055/s-0030-1269908

[11] Sinove Y Kyriopoulos E Ceulemans P Houtmeyers P Hoebeke P Monstrey S. Preoperative planning of a pedicled anterolateral thigh (ALT) flap for penile reconstruction with the multidetector CT scan. Handchir Mikrochir Plast Chir. 2013;45:217–222. 23468232 10.1055/s-0032-1333271

[12] Liu CY Wei ZR Jiang H Zhao Y-Z Zhang Y-F. Preconstruction of the pars pendulans urethrae for phalloplasty with digestive mucosa using a prefabricated anterolateral thigh flap in a one-arm patient. Plast Reconstr Surg Glob Open. 2013;1:e53. 25289248 10.1097/GOX.0b013e3182aa8779 PMC4174055

[13] Peters BR Richards HW Berli JU. Optimizing innervation in radial forearm phalloplasty: consider the posterior antebrachial cutaneous nerve. Plast Reconstr Surg. 2023;151:202–206. 36576827 10.1097/PRS.0000000000009771

[14] D’Arpa S Claes K Lumen N Oieni S Hoebeke P Monstrey S. Urethral reconstruction in anterolateral thigh flap phalloplasty: a 93-case experience. Plast Reconstr Surg. 2019;143:382e–392e. 10.1097/PRS.0000000000005278 30688908

[15] Monstrey SJ Ceulemans P Hoebeke P. Sex reassignment surgery in the female-to-male transsexual. Semin Plast Surg. 2011;25:229–244. 22851915 10.1055/s-0031-1281493 PMC3312187

[16] Koshima I Nanba Y Nagai A Nakatsuka M Sato T Kuroda S. Penile reconstruction with bilateral superficial circumflex iliac artery perforator (SCIP) flaps. J Reconstr Microsurg. 2006;22:137–142. 16780040 10.1055/s-2006-939957

[17] Perović S. Phalloplasty in children and adolescents using the extended pedicle island groin flap. J Urol. 1995;154:848–853. 7609196 10.1097/00005392-199508000-00142

[18] Aköz T Erdoğan B Görgü M Kapucu MR Kargi E. Penile reconstruction in children using a double vascular pedicle composite groin flap. Scand J Urol Nephrol. 1998;32:225–230. 9689706 10.1080/003655998750015647

[19] Zieliński T. Phalloplasty using a lateral groin flap in female-to-male transsexuals. Acta Chir Plast. 1999;41:15–19. 10394175

[20] World Professional Association of Transgender Health. Standards of Care Version 7. Available at: https://wpath.org. Accessed March 31, 2025.

[21] Whitman PA Adigun OO. Anatomy, Skin, Dermatomes. In: StatPearls. Treasure Island, FL: StatPearls Publishing; 2023. 30571022

[22] Sommeling CE De Wolf EJ Salim A. A new technique for coronaplasty in penile reconstruction. J Sex Med. 2018;15:920–923. 29501425 10.1016/j.jsxm.2018.01.024

[23] Hage JJ Bouman F de Graaf F Bloem JJAM. Construction of the neophallus in female-to-male transsexuals: the Amsterdam experience. J Urol. 1993;149:1463–1468. 8501789 10.1016/s0022-5347(17)36416-9

[24] Salgado CJ Chim H Tang JC Monstrey SJ Mardini S. Penile reconstruction. Semin Plast Surg. 2011;25:221–228. 22851914 10.1055/s-0031-1281492 PMC3312184

[25] Selvaggi G Bellringer J. Gender reassignment surgery: an overview. Nat Rev Urol. 2011;8:274–282. 21487386 10.1038/nrurol.2011.46

[26] Santucci RA. Urethral complications after transgender phalloplasty: strategies to treat them and minimize their occurrence. Clin Anat. 2018;31:187–190. 29178533 10.1002/ca.23021

[27] Schaff J Papadopulos NA. A new protocol for complete phalloplasty with free sensate and prelaminated osteofasciocutaneous flaps: experience in 37 patients. Microsurgery. 2009;29:413–419. 19399883 10.1002/micr.20647

[28] Rohrmann D Jakse G. Urethroplasty in female-to-male transsexuals. Eur Urol. 2003;44:611–614. 14572764 10.1016/s0302-2838(03)00356-7

[29] Morrison SD Massie JP Dellon AL. Genital sensibility in the neophallus: getting a sense of the current literature and techniques. J Reconstr Microsurg. 2018;35:129–137. 30078177 10.1055/s-0038-1667360

[30] Kaur MN Morrison SD Kennedy SL. International study to develop a patient-reported outcome measure to evaluate outcomes of gender-affirming care - the GENDER-Q. J Patient Rep Outcomes. 2024;8:134. 39560846 10.1186/s41687-024-00785-x PMC11576686

