# Supplementary figures and images for: Use of the Superficial Circumflex Iliac Artery Perforator Flap for Urethra and/or Shaft Reconstruction in Gender-Diverse Persons: 10-Year Single-Center Experience
Source: Plast Reconstr Surg. 2024 Oct 28;155(6):1036–44. doi: 10.1097/PRS.0000000000011830 (PMC12105959; doi:10.1097/PRS.0000000000011830)

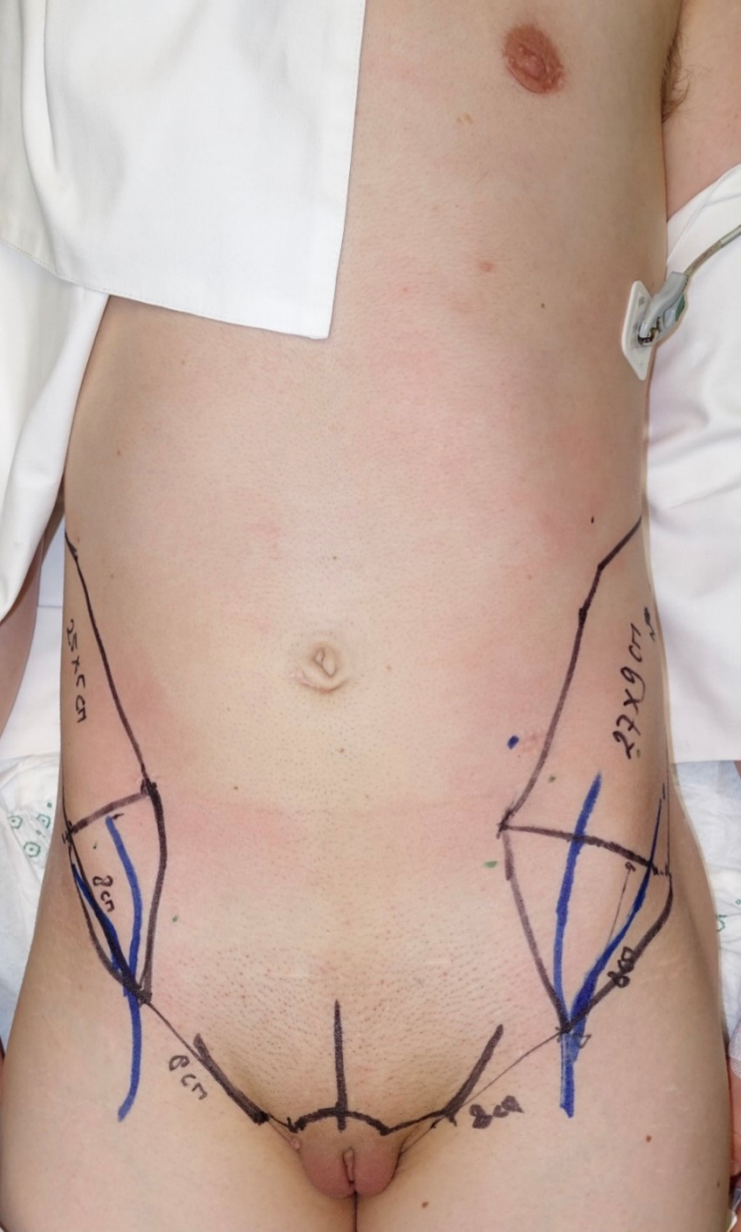

Supplement: Supplementary file 1 [file prs-155-1036e-s001.pdf]

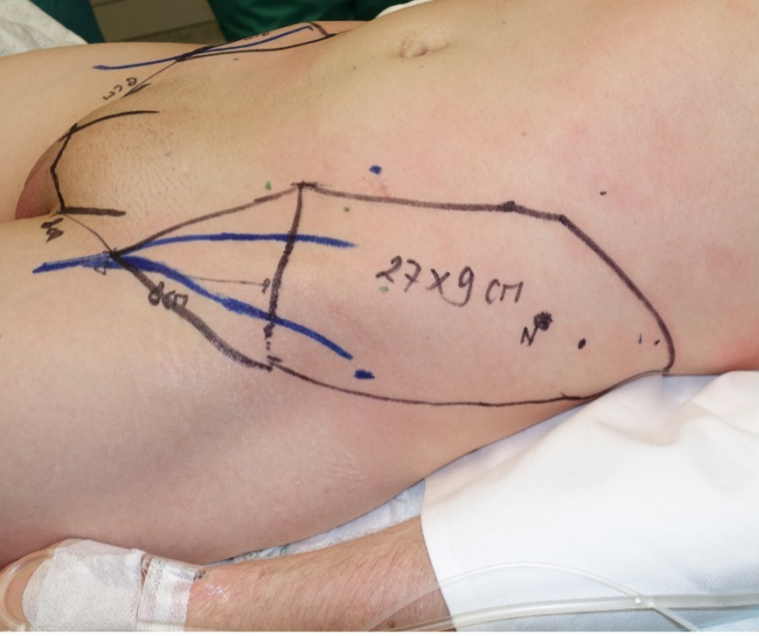

Supplement: Supplementary file 2 [file prs-155-1036e-s002.pdf]

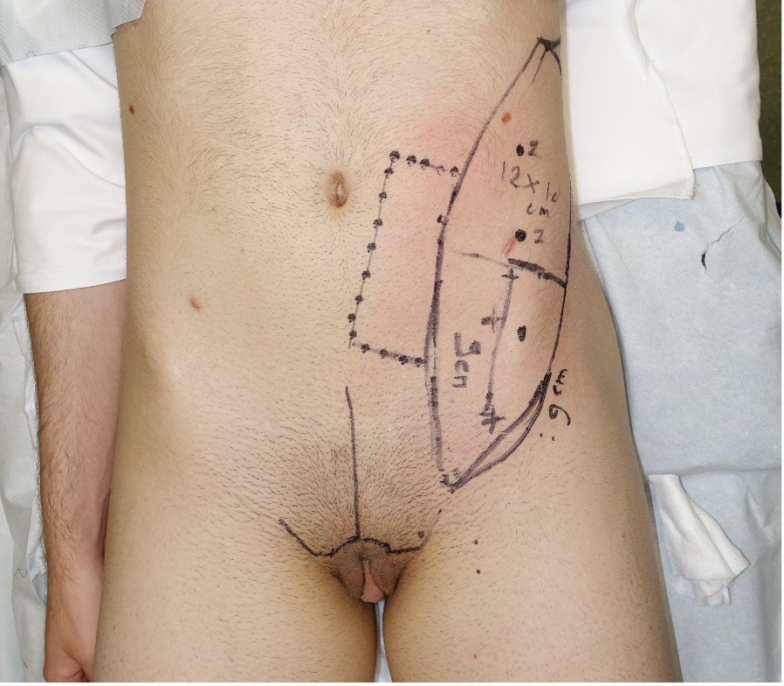

Supplement: Supplementary file 3 [file prs-155-1036e-s003.pdf]
